# Young male sex workers are at high risk for sexually transmitted infections, a cross-sectional study from Dutch STI clinics, the Netherlands, 2006–2012

**DOI:** 10.1186/s12879-016-1388-3

**Published:** 2016-02-04

**Authors:** N. Fournet, F. D. H. Koedijk, A. P. van Leeuwen, M. S. van Rooijen, M. A. B. van der Sande, M. G. van Veen

**Affiliations:** 1 Epidemiology and Surveillance Unit, Centre for Infectious Diseases Control, National Institute for Public Health and the Environment (RIVM), P.O. Box 1, 3720 BA Bilthoven, The Netherlands; 2 European Programme for Intervention Epidemiology Training (EPIET), European Centre for Disease Prevention and Control (ECDC), Stockholm, Sweden; 3 Public Health Service Twente, Enschede, The Netherlands; 4 Public Health Service Amsterdam, Amsterdam, The Netherlands; 5 Julius Centre for Health Sciences and Primary Care, University Medical Centre Utrecht, Utrecht, The Netherlands

**Keywords:** Sexually transmitted infections, Sex workers, Public health surveillance, Male, HIV, Risk factors

## Abstract

**Background:**

Male sex workers (MSW) are particularly exposed to sexually transmitted infections (STI) including HIV. In the Netherlands, data about STI among MSW are scarce. We estimated chlamydia, gonorrhoea, syphilis and HIV diagnoses among MSW attending STI clinics and determined associated factors to guide prevention policies.

**Methods:**

Using 2006–2012 cross-sectional national surveillance data from Dutch STI clinics, we calculated the proportion of consultations with a positive test for any of three bacterial STI or HIV among MSW. Associated factors were determined by using Poisson logistic regression with robust variance.

**Results:**

We identified 3,053 consultations involving MSW, of which 18.1 % included at least one positive bacterial STI test and 2.5 % a positive HIV test. Factors associated with bacterial STI and/or HIV diagnoses were respectively age groups < 35 y.o. and self-reporting homo- or bisexual preferences (aRR = 1.6; 95 % CI: 1.3–2.1), and age group 25–34 y.o. (aRR = 2.7; 95 % CI: 1.2–6.5) and self-reporting homo- or bisexual preferences (aRR = 24.4; 95 % CI: 3.4–176.9). Newly diagnosed and pre-existing HIV infection were associated with an increased risk for bacterial STI (aRR = 2.7, 95 % CI: 1.7–2.6 and aRR = 2.1, 95 % CI: 2.2–3.4 respectively). MSW with no history of HIV screening were more likely to be tested positive for HIV compared to those with a previous HIV-negative test (aRR = 2.6, 95 % CI: 1.6–4.3).

**Conclusion:**

Health promotion activities should target MSW who are young, homo- or bisexual, those who are HIV-infected or who have never been tested for HIV, to increase early diagnosis, prevention and treatment.

## Background

Sex workers are at high risk for contracting sexually transmitted infections (STI) including human immunodeficiency virus (HIV) [1–3]. In Europe, the majority of sex workers are women whereas male sex workers (MSW) represent an estimated 7 % of the sex workers population [4]. Studies about risk for acquiring bacterial STI and HIV among MSW are limited but suggest a high risk among this population, even higher than among female sex workers (FSW) [5, 6].

A European HIV/AIDS survey reported a high prevalence of HIV among non-injecting drug-using (IDU) MSW (up to 12 % in Spain) compared to less than 1 % among non-IDU FSW [7]. Studies in Belgium [6] and Australia [3] reported that almost one-third of MSW had one or more STI. These higher prevalences of STI and HIV among MSW than among FSW can be explained by differences in sexual behaviours and characteristics. Indeed, more than 85 % of MSW have sex with men [3, 5, 8–10]. Men who have sex with men (MSM) have been reported to more frequently engage in unsafe sex practices [10] and to have higher prevalences of STI and HIV compared to heterosexual men [11–14]. Furthermore, the male sex work industry seems markedly different from the female sex work sector. It is less organised and MSW tend to hide their commercial sexual practices [5, 15]. In a study on MSW attending genitourinary clinics in the United Kingdom in 2011, MSW more frequently attended clinics and were significantly more likely to be diagnosed with a bacterial STI or with HIV than other male attendees [16]. Additional studies suggest that MSW are difficult to reach by education and intervention programmes [5, 15].

In the majority of European countries, sex work is prohibited, although largely tolerated. In the Netherlands, however, voluntary adult prostitution is recognised as a legal occupation. In 2000, a national law was passed to allow, regulate, and control prostitution in which adult sex workers are voluntarily engaged. In 2004, a study reported an estimated 25,000 sex workers in the country, 90 % being women, 5 % men and 5 % transgender [17]. Due to the high mobility of sex workers, absolute numbers could be underestimated.

In the Netherlands, many studies about STI among high risk groups concerned MSM [11, 13, 18–20], FSW, and transgender sex workers [21], but so far none focused on men, including MSM, involved in the sex work industry. Therefore, a sound understanding of MSW characteristics is needed to develop comprehensive sexual health promotion programmes targeting this group.

The first objective of this study was to assess the percentage of consultations with at least one positive bacterial (chlamydia, gonorrhoea, syphilis) STI or HIV test among MSW who attended STI clinics in the Netherlands between 2006 and 2012. The second objective was to determine factors associated with bacterial STI and HIV diagnoses among MSW to guide targeted prevention programmes.

## Method

### Setting

In the Netherlands, 26 STI clinics — mostly within the Public Health Services — are distributed across the country and provide anonymous free-of-charge STI/HIV testing and treatment of STI for high risk groups. High risk groups encompass people matching one or more of the following criteria: reporting STI-related symptoms, notified or referred for STI testing, aged below 25 years, MSM, involved in sex work, originated from HIV endemic area, reporting three or more sexual partners in the previous six months or reporting a partner from one of these high risk groups.

At STI clinics all attendees are anonymously tested for chlamydia, gonorrhoea, and syphilis even if the individual do not present symptoms or complaints. Diagnoses are carried out locally in STI clinics-affiliated laboratories in accordance with standard procedures [14]. Chlamydia diagnosis is performed in all laboratories by using nucleic acid amplified test (NAAT) on urine sample or urethral swab*. Neisseria gonorrhoea* diagnosis methods vary between laboratories: culture is primarily performed in symptomatic attendees whereas NAAT is primarily performed in asymptomatic attendees. Syphilis testing is done using *Treponema pallidum* hemagglutination assay (TPHA) [14]. Since 2010, a national opt-out policy for HIV testing is implemented and HIV tests are routinely performed at each visit unless the patient refuses by using previously described methods [22, 23]. Dutch guidelines for MSW recommend to test for STI and HIV every 3 months [24]. Since 2004, demographic, behavioural and clinical information are recorded by physician or nurse in an online registration surveillance database and reported to the Centre for Infectious Diseases Control at the National Institute for Public Health and the Environment (RIVM).

Registration in STI clinics has been harmonised in 2006, therefore, the 2006–2012 period was selected to allow comparable data between the 26 STI clinics.

Ethical approval for the study was not necessary following Dutch law as the study used anonymous patient data collected for routine surveillance [25].

### Study design

We conducted a cross-sectional study using all consultations of MSW registered in the online RIVM registration database from 2006 to 2012. Because of the anonymous nature of this surveillance database, identification of repeated consultations for a defined individual was not possible. Consequently, the unit of analysis was a consultation for which a test for bacterial STI or HIV was performed.

### Study population

We defined a MSW as a man who reports the exchange of sex for money or other valuable goods, such as drugs, and who has been involved in sex work (legally or not) at least once in the six months prior to consultation at the STI clinic.

### Outcomes

For each consultation, two clinical outcomes were analysed separately:

- positive test result for bacterial STI (chlamydia, gonorrhoea, and/or syphilis) and
- positive test result for HIV

We determined the proportion of consultations with at least one positive test result for either a bacterial STI or HIV among MSW.

### Data collection and statistical analysis

For each STI clinic visit, data recorded in the national database included year and area of origin, gender, self-defined sexual preference (hetero-, homo- or bisexual), injecting drug use in the past six months (yes/no), bacterial (chlamydia, gonorrhoea or syphilis) STI in the past two years (yes/no), previous HIV test (no test/positive test/negative test) and clinical outcomes. Age groups 15–24 years, 25–34 years and ≥35 years were analysed and sexual preferences were analysed in two categories: homo/bisexual.

Descriptive analyses were performed for demographic, behavioural, and clinical data. Trends between 2006 and 2012 were determined using the Cochran-Armitage trend test.

Analyses were conducted using SAS 9.3 (SAS Institute Inc., Cary, North Carolina, USA).

Factors associated with a positive test result for either a bacterial STI or for HIV among MSW were determined by using a Poisson logistic regression model with robust variance. Variables associated with the outcomes in univariate analysis (*p*-value < 0.20) were included in a multivariate model and variables with a *p*-value greater than 0.05 were eliminated step-by-step, controlling for confounding at each step. Interaction effects were tested in the final model. Results of univariate and multivariate statistical analyses were expressed as adjusted risk ratios (aRR) with Wald 95 % confidence intervals.

For bacterial STI outcome, we studied the association with HIV status considering the following three modalities collected during consultation: negative HIV test/new positive HIV diagnosis/known HIV infection. For new HIV diagnosis outcome, the information about previous HIV tests (yes/no) and co-infection with a bacterial STI (yes/no) diagnosed at the current consultation were included.

## Results

### Characteristics of MSW

Of the 33,719 consultations in Dutch STI clinics involving sex workers between 2006 and 2012, 90.5 % involved FSW, 9.1 % involved MSW and 0.4 % involved transgender. Among the 3,053 consultations involving MSW, median age was 30 years (interquartile range: 24–38 years) and 50 % were from the Netherlands (Table 1). Homo- or bisexual preference, history of bacterial STI in the past two years, and injecting drug use in the past 6 months were respectively reported in 72, 16 and 2 % of consultations involving MSW. History of previous HIV testing was reported in 78 % of consultations involving MSW and a positive HIV test result was reported in 9 % of previously tested MSW.

**Table 1:** Characteristics of MSW attending STI clinics in the Netherlands, 2006–2012

	MSW (*N* = 3,053)	
	Number^a^	%
Age group		
15–24 years	789	26
25–34 years	1,252	41
≥ 35 years	1,011	33
Country/Area of origin		
The Netherlands	1,511	50
Eastern Europe	620	20
Other European countries	134	4
Latin America	437	14
Sub-Saharan Africa	40	1
Asia	57	2
Turkey/Morocca	127	4
Other or unknown	124	4
Self-defined sexual preference		
Heterosexual	855	28
Homo/bisexual	2,183	72
Intravenous drug user in the past 6 months		
No	2,919	98
Yes	64	2
Previous bacterial STI^b^ in the past 2 years		
No	2,208	84
Yes	429	16
Previous HIV test		
No	648	22
Yes	2,345	78
HIV result among MSW previously tested		
Positive test	207	9
Negative test	2,138	91

^a^Missing data: 1 for age, 3 for Country/area of origin, 15 for self-defined sexual preference, 70 for intravenous drug use, 416 for previous bacterial STI, 60 for previous HIV test

^b^Chlamydia, gonorrhoea and/or syphilis

### Percentage of consultations with a positive bacterial STI or HIV test

Between 2006 and 2012, the number of consultations among MSW attending STI clinics for bacterial STI and HIV testing respectively rose from 270 to 663 and from 196 to 599.

**Fig. 1:**
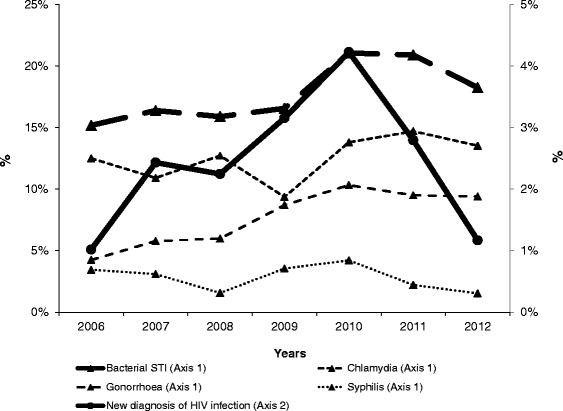
Percentage of consultations with a positive bacterial STI test or positive HIV test among MSW at STI clinics in the Netherlands, 2006–2012

The percentage of positive bacterial STI tests increased from 15.2 % in 2006 to 21.1 % in 2010, remained stable until 2011, and dropped to 18.3 % in 2012 (Fig. 1). The Cochran-Armitage trend test indicated an increasing trend in the percentage of positive bacterial STI (*p* = 0.017). This trend is primarily related to an increase in chlamydia (from 12.5 to 13.5 % between 2006 and 2012) and gonorrhoea (from 4.3 to 9.4 %) diagnoses whereas syphilis remained stable around 3 %. Over the study period, the percentage of positive bacterial STI tests was 18.1 % (95 % CI: 16.7–19.5) among 3,016 consultations involving MSW tested for bacterial STI: one, two (mostly chlamydia and gonorrhoea) and three concurrent STI were respectively confirmed in 15.1, 2.9 and 0.2 % of these consultations. In total, chlamydia was reported in 11.3 % of the consultations, gonorrhoea in 7.5 % and syphilis in 2.6 %.

Between 2006 and 2012, the percentage of positive HIV tests was 2.5 % (95 % CI: 1.9–3.0) among 2,688 consultations involving MSW tested for HIV.

As shown in Fig. 1, the percentage of positive HIV tests over time followed the same trend as those for bacterial STI: it increased from 1.0 % in 2006 to 4.2 % in 2010 then decreased to 1.2 % in 2012, however, this trend was not significant (*p* = 0.4).

### Factors associated with a positive bacterial STI test

In the multivariate analysis (Table 2), adjusted on significant variables, compared to MSW older than 35 years, younger MSW were more likely to be diagnosed with a bacterial STI: 2.3 times more for MSW between 15 and 24 years old and 1.4 times more for MSW between 25 and 34 years (*p* < 0.001). MSW who defined themselves as homo-or bisexual had a 62 % increased risk to have a positive bacterial STI test than heterosexual MSW (aRR = 1.62; 95 % CI: 1.27–2.06). Reporting a history of bacterial STI in the past two years was associated with an increased risk of 39 % of having a bacterial STI at the current consultation (aRR = 1.39; 95 % CI: 1.15–1.68). Compared to MSW with a negative HIV test at the current consultation, MSW who had a positive HIV test had a 2.7 higher risk of having a bacterial STI (aRR = 2.71; 95 % CI: 1.68–2.64) and those with a known HIV-positive status had a 2.1 higher risk (aRR = 2.11; 95 % CI: 2.15–3.43).

**Table 2:** Factors associated with at least one positive bacterial STI test for chlamydia, gonorrhoea and/or syphilis in consultations involving MSW (*N* = 3,016) at STI clinics — the Netherlands, 2006–2012

	Diagnosed^a^ (*n*)	Tested^a^ (*n*)	Percent	Univariate analysis			Multivariate analysis^b^		
				RR	95 % CI	*p* value*	RR adj^c^	95 % CI	*p* value*
Age group						**<.001**			**<.001**
≥ 35 years	119	1 000	11.9	ref			ref		
25–34 years	220	1 238	17.8	1.49	1.21–1.84	**<.001**	1.41	12–1.77	**0.003**
15–24 years	208	777	26.8	2.25	1.84–2.76	**<.001**	2.30	1.83–2.88	**<.001**
Country/Area of origin						0.06			
The Netherlands	245	1 478	16.6	ref					
Eastern Europe	129	618	20.9	1.26	1.04–1.52	0.02			
Other European countries	29	133	21.8	1.32	0.93–1.85	0.12			
Latin America	75	436	17.2	1.04	0.82–1.31	0.76			
Sub-Saharan Africa	4	40	10.0	0.60	0.24–1.54	0.29			
Asia	12	57	21.1	1.27	0.76–2.13	0.36			
Turkey/Morocco	32	127	25.2	1.52	1.10–2.09	0.01			
Other or unknown	21	124	16.9	1.02	0.68–1.53	0.92			
Self-defined sexual preference									
Heterosexual	87	839	10.4	ref			ref		
Homosexual/Bisexual	456	2 162	21.1	2.03	1.64–2.52	**<.001**	**1.62**	**1.27–2.06**	**<.001**
Intravenous drug user in the past 6 months									
No	524	2 886	18.2	ref					
Yes	10	62	16.1	0.89	0.50–1.58	0.69			
Previous bacterial STI^d^ in the past 2 years									
No	364	2 181	16.7	ref			ref		
Yes	112	421	26.6	1.59	1.33–1.92	**<.001**	**1.39**	**1.15–1.68**	**0.001**
HIV test result						**<.001**			**<.001**
Negative test	415	2 611	15.9	ref			ref		
Positive test	37	65	56.9	3.58	2.85–4.50	**<.001**	**2.71**	**1.68–2.64**	**<.001**
Known HIV positive	76	205	37.1	2.33	1.91–2.84	**<.001**	**2.11**	**2.15–3.43**	**<.001**

^*^Bold numbers are significant p value <0.05

^a^509 missing data out of a total of 3,016 consultations in MSW (17 %): 1 for age, 3 for country/area of origin, 15 for self-defined sexual preference, 68 for injecting drug use, 414 for previous bacterial STI, 135 for previous HIV test

^b^Variables included in the multivariate analysis: age group, self-defined sexual preference, previous bacterial STI and HIV test result

^c^Adjusted for age group, self-defined sexual preference, previous bacterial STI and HIV test

^d^Chlamydia, gonorrhoea and/or syphilis

### Factors associated with a positive HIV test

In the multivariate analysis, MSW aged between 25 and 34 years were 2.7 more likely to have a positive HIV test than MSW older than 35 years (aRR = 2.74; 95 % CI: 1.15–6.50). MSW who defined themselves to be homo- or bisexual were 24.4 times more likely to be tested positive for HIV than heterosexual MSW (aRR = 24.41; 95 % CI: 3.37–176.88). MSW who were diagnosed with a bacterial STI had a 384 % increased risk to be also diagnosed with HIV than those who tested negative for bacterial STI (aRR = 4.84; 95 % CI: 2.96–7.90). MSW who were never tested for HIV prior to their consultation were 2.6 times more likely to have a positive HIV test than those who had previously been tested negative for HIV (aRR = 2.59; 95 % CI: 1.56–4.29) (Table 3).

**Table 3:** Factors associated with a positive HIV test in consultations involving MSW (*N* = 2,688) at STI clinics —the Netherlands, 2006–2012

	Diagnosed^a^ (*n*)	Tested^a^ (*n*)	Percent	Univariate analysis			Multivariate analysis^b^		
				RR	95 % CI	*p* value*	RR adj^c^	95 % CI	*p* value*
Age group						**<0.001**			**<0.001**
≥ 35 years	6	859	0.7	ref			ref		
25–34 years	29	1 087	2.7	3.82	1.59–9.16	**0.003**	2.74	1.15–6.50	**0.02**
15–24 years	31	742	4.2	5.98	2.51–14.26	**<0.001**	2.43	0.99–5.92	0.05
Country/Area of origin						0.07			
The Netherlands	24	1 297	1.9	ref					
Eastern Europe	18	587	3.1	1.66	0.91–3.03	0.10			
Other European countries	6	118	5.1	2.75	1.14–6.59	0.02			
Latin America	13	387	3.4	1.82	0.93–3.53	0.08			
Sub-Saharan Africa	2	37	5.4	2.92	0.72–11.91	0.14			
Asia	0	46	0.0	–					
Turkey/Morocco	3	116	2.6	1.40	0.43–4.57	0.58			
Other or unknown	0	97	0.0	–					
Self-defined sexual preference									
Heterosexual	1	793	0.1	ref			ref		
Homosexual/Bisexual	64	1 881	3.4	26.98	3.75–194.21	**<0.001**	24.41	3.37–176.88	**0.002**
Intravenous drug user in the past 6 months									
No	66	2 585	2.6						
Yes	0	52	0.0						
Previous bacterial STI^d^ in the past 2 years									
No	51	1 999	2.6	ref					
Yes	12	344	3.5	1.37	0.74–2.54	0.32			
Co-infection with a bacterial STI									
No	28	2 224	1.3	ref			ref		
Yes	37	452	8.2	6.50	4.02–10.51	**<0.001**	4.84	2.96–7.90	**<0.001**
Previous HIV test result									
Negative test	42	2 060	2.0	ref			ref		
No test	21	576	3.6	1.79	1.07–2.99	**0.03**	2.59	1.56–4.29	**<0.001**

^*^Bold numbers are significant p value <0.05

^a^77 missing data out of a total of 2688 consultations in MSW (3 %): 3 for country/area of origin, 14 for self- defined sexual preference, 51 for injecting drug use, 345 for previous bacterial STI, 12 for STI co-infection, 52 for previous HIV test

^b^Variables included in the multivariate analysis: age group, self- defined sexual preference, co-infection with bacterial STI and previous HIV test result

^c^Adjusted for age group, self- defined sexual preference, co-infection with bacterial STI and previous HIV test result

^d^Chlamydia, gonorrhoea and/or syphilis

## Discussion

Between 2006 and 2012, 18.1 % of consultations at Dutch STI clinics involving MSW resulted in a positive bacterial STI test, and 2.5 % in a positive HIV test. Factors associated with either a positive bacterial STI or HIV test were young age groups and reporting homo- or bisexual preferences. HIV-positive (new diagnosis or previously known status) MSW were more likely to be diagnosed with a bacterial STI, and those who had never been tested for HIV prior to their consultation were at higher risk for having a positive HIV test than those who previously tested negative.

Limited studies have examined STI and associated risk factors among MSW in the Netherlands. Our results are consistent with other studies performed in Europe among MSW [5, 6, 9] and confirm that MSW are an important population to target for STI control strategies. However, results have to be interpreted and compared with other studies with caution because our data did not allow identification of repeated consultations for a defined individual.

In our study, median age of MSW was 29 years and 33 % were older than 35 years, which is consistent with a study conducted among MSW attendees in STI clinics in the United Kingdom in 2011 [16]. Other studies conducted before 2004 [3, 6, 10] report a lower median age (between 25 and 27). These differences could be explained by changes in MSW characteristics over time. Mean age of MSW visiting STI clinics could have increased over time because MSW could be older or older MSW could visit more often STI clinic.

Our results indicate that the percentage of consultations with either a positive STI or HIV test is significantly higher among younger MSW than among those older than 35 years. In similar studies conducted before 2002 [3, 6, 10], age was not significantly associated with a high risk of STI. Use of safer sexual practices among older MSW might explain these discrepancies over time.

The percentages of consultations with bacterial STI and HIV diagnosis were higher among MSW who defined themselves as homo- or bisexual than among heterosexual. The same result is found among all consultations in STI clinics in the Netherlands between heterosexual males and MSM [26]. Moreover, the percentage of consultations with a positive bacterial STI or HIV test among young MSW who defined themselves as homo- or bisexual (27 and 4 %, respectively for bacterial STI and HIV) is higher than the prevalence reported in 2012 among young MSM (20 %, *p* = 0.01 and 1 %, *p* = 0.01) who visited an STI clinic [26]. In addition, an increased risk for both STI and HIV infection associated with more frequent unsafe sexual practices have also been reported among MSM compared to heterosexuals [12–14], as well as a higher HIV seroprevalence among MSM sex workers [5, 27].

We found a high percentage of consultations with co-infections: HIV seropositivity (newly diagnosed or previously known status) was highly associated with bacterial STI. This result is also consistent with previous studies [12, 28].

In another study implemented in the Netherlands among FSW between 2002 and 2005, HIV prevalence was found to be 1.5 %. With a percentage of positive HIV tests of 2.5 %, our study suggest that MSW are more at risk for HIV than FSW [7].

Our study has several limitations. Firstly, the database does not allow identification of repeated consultations for a defined person. Having repetitive consultations could be an indicator of higher risk of infection if MSW attend the clinic because of persistent risk behaviour or if having symptoms. In a UK study implemented in STI clinics in 2011 [16], MSW had a higher average number of visits than other male attendees and were more likely to be diagnosed with HIV, chlamydia or gonorrhoea and experience reinfections. Moreover, because identification of repeated consultations was not possible, we were not able to calculate incidence which is a more relevant and accurate indicator than the percentage of consultation with positive bacterial or HIV tests.

Secondly, STI clinic used different variable laboratory testing methods and that may have contributed to differences in the detection of STI diagnosis.

Another limitation is that MSW attending STI clinics may not be representative of the overall MSW population in the Netherlands. In addition to STI clinics, STI healthcare in the Netherlands is also provided by general practitioners, HIV treatment centres, and specialised hospital facilities [29]. Furthermore, the most marginalised MSW, those performing illegal business or with an illegal status, may not attend STI clinics or other healthcare providers. Therefore, factors associated with bacterial STI or HIV infection in our study may not be generalizable.

Finally, other factors known to be associated with a higher risk for STI among sex workers, such as sexual practices, condom use, recruitment area, sexual techniques with clients, number of partners, steady or casual partners, etc., were not available in the surveillance database therefore we were not able to investigate more in depth the risk factors associated with both bacterial STI and HIV infection among MSW. Sexual practices and condom use are recorded by some STI clinics but data are not recorded in the national database, which could be improved in the future to allow more in-depth researches.

## Conclusion

In conclusion, the high percentage of MSW consultations at Dutch STI clinics with either a positive bacterial STI or HIV test confirms that MSW represent a high risk group for STI infections. MSW, particularly those who have sex with men, are at high risk of contracting STI including HIV and therefore may transmit these infections to their clients or partners. Prevention and intervention activities should particularly target MSW who engage in homo- or bisexual intercourse to stimulate HIV testing, increase early STI diagnoses, ensure early treatment, and therefore interrupt further transmission. In this hard-to-reach population, the Internet could be a useful tool to enhance reach and implementation of actions. Additional studies are needed to investigate other risk factors, identify opportunities for interventions, target MSW populations who do not visit STI clinics and evaluate the impact of interventions measures in this group.

## Abbreviations

CI: confident interval
ECDC: European Centre for Disease Prevention and Control
EPIET: European Programme for Intervention Epidemiology Training
HIV: human immunodeficiency virus
MSW: male sex worker
RR: risk ratio
RIVM: National Institute for Public Health and the Environment
STI: sexually transmitted infections
SW: sex worker
